# Acquiesced First Sex and Sexual Quality in Older Adults

**DOI:** 10.51894/001c.143899

**Published:** 2025-09-30

**Authors:** Megan Russ

**Affiliations:** 1 College of Osteopathic Medicine Michigan State University

## 14

Despite growing scholarly attention to the importance of sexual health in older adults, little is known about the ways in which early sexual experiences shape sexual behavior and well-being in later life. This study identifies acquiescence – or participation in sex without concomitant desire – at sexual debut as one such factor that may contribute to sexual dysfunction in older adulthood. Utilizing survey data from the National Social Life, Health, and Aging Project (NSHAP), the study examines the relationship between acquiesced first sex and sexual quality, including both physical pleasure and emotional satisfaction, in a sample of sexually active older adults (n=1780). Ordered logistic regression and tests of predicted probabilities were used to analyze both the main effect of first-sex acquiescence and potential interaction effects with gender. The study finds that acquiesced first sex is associated with lower levels of both physical pleasure (OR = 0.67, p < 0.01) and emotional satisfaction (OR = 0.66, p < 0.01) with sex, and that the deleterious effects of acquiescence on physical pleasure are stronger in female respondents than in males. These results bring attention to the importance of a complete sexual history in physicians’ understandings of sexual dysfunction and long-term sexual well-being. Additionally, findings suggest that educating adolescents on sexual consent and healthy sexual boundaries to prevent experiences of acquiesced first sex may be a point of intervention for improving sexual wellness throughout the life course.

